# Co-morbid tics and stereotypies: a systematic literature review

**DOI:** 10.1007/s10072-023-07095-y

**Published:** 2023-09-29

**Authors:** Andrea E. Cavanna, Giulia Purpura, Anna Riva, Renata Nacinovich

**Affiliations:** 1 Department of Neuropsychiatry, National Centre for Mental Health, BSMHFT and University of Birmingham, 25 Vincent Drive, Birmingham, B15 2FG UK; 2 Sobell Department of Motor Neuroscience and Movement Disorders, Institute of Neurology and University College London, London, UK; 3 School of Life and Health Sciences, Aston Brain Centre, Aston University, Birmingham, UK; 4 School of Medicine and Surgery, University of Milano-Bicocca, Milan, Italy; 5 Department of Child Neuropsychiatry, IRCCS San Gerardo dei Tintori, Monza, Italy

**Keywords:** Hyperkinetic movement disorders, Stereotypies, Tics, Tourette syndrome

## Abstract

**Background:**

Tics and stereotypies are childhood-onset repetitive behaviours that can pose significant diagnostic challenges in clinical practice. Both tics and stereotypies are characterised by a complex co-morbidity profile, however little is known about the co-occurrence of these hyperkinetic disorders in the same patient population.

**Objective:**

This review aimed to assess the relationship between tics and stereotypies when these conditions present in co-morbidity.

**Methods:**

We conducted a systematic literature review of original studies on co-morbid tics and stereotypies, according to the standards outlined in the Preferred Reporting Items for Systematic Reviews and Meta-Analyses (PRISMA) guidelines.

**Results:**

Our literature search identified six studies of suitable sample size (*n* ≥ 40) presenting data on the association between tics and stereotypies in otherwise typically developing patients. A considerable proportion (23%) of patients diagnosed with stereotypic movement disorder present with co-morbid tics (range 18–43%). Likewise, the prevalence of primary stereotypies is increased in patients with tic disorders such as Tourette syndrome (8%, range 6–12%).

**Discussion:**

Tics and stereotypies can often develop in co-morbidity. The association of tics and stereotypies in the same patient has practical implications, in consideration of the different treatment approaches. Future research should focus on the assessment and management of both conditions, particularly in special populations (e.g. patients with pervasive developmental disorders).

## Introduction

Tics and stereotypies are the most common hyperkinetic movement disorders occurring during the developmental period [1]. The current edition of the DSM [2] lists tic disorders and stereotypic movement disorder alongside developmental coordination disorder as motor disorders within the category of neurodevelopmental conditions [2]. Tics are defined as sudden, rapid, recurrent, non-rhythmic movements or vocalizations, such as eye blinking, grimacing, shoulder shrugging, grunting and sniffing [3]. Tics can be either transient (as in a proportion of young patients initially diagnosed with a provisional tic disorder) or chronic (as in patients with a persistent motor/vocal tic disorder). Tourette syndrome is the most complex chronic tic disorder, as its diagnosis requires the presence of both multiple motor tics and at least one vocal tics, with onset in the paediatric age [2]. Stereotypies are defined as repetitive, seemingly driven, and apparently purposeless motor behaviours (e.g. hand flapping or waving, body rocking, head banging, self-biting or hitting), with onset in the early developmental period [4, 5]. Only subjects whose repetitive motor behaviours interfere with social, academic, or other activities (or result in self-injury) fulfil current diagnostic criteria for stereotypic movement disorder [2].

The differential diagnosis between tics and stereotypies can pose significant challenges, in consideration of their clinical and pathophysiological overlap [6]. Specifically, alterations at the level of cortico-basal ganglia circuitries have been shown to be associated with the expression of repetitive behaviours, particularly in male subjects [1]. From a clinical perspective, tics and stereotypies can have different localisation, association with sensory manifestations, time course, and relationship with environmental triggers. Stereotypic movements tend to have a more complex and rhythmic nature than tics, with predominant involvement of the limbs or trunk [1]. Conversely, tics develop most commonly at the level of the cephalic district and are characteristically associated with distressing sensations of increased inner tension, referred to as premonitory urges [7, 8]. Stereotypies are often triggered by excitement and self-stimulation, whereas tics typically follow a waxing and waning course, with possible exacerbations concomitant to increased underlying anxiety [1]. Moreover, in most cases stereotypies have an earlier age of onset than tics (as early as 3 years *versus* 5–7 years) [4, 5]. Establishing the correct diagnosis is of paramount importance for the implementation of psychoeducation and targeted treatment interventions, which are indicated whenever the hyperkinetic manifestations are severe enough to cause distress. Beyond behavioural strategies, the evidence base for the efficacy of pharmacotherapy is considerably stronger for tics compared to stereotypies [9, 10].

Both tics and stereotypies are characterised by a complex co-morbidity profile, with increased prevalence in subjects with other neurodevelopmental conditions (autism spectrum disorder, attention-deficit and hyperactivity disorder) [11, 12]. Tics are often accompanied by specific obsessive–compulsive behaviours, as well as anxiety and affective symptoms, especially in the setting of Tourette syndrome and other neurodevelopmental disorders [13, 14]. Compared to tics, stereotypies are more likely to be associated with developmental coordination difficulties [15]. Little is known about the co-occurrence of tics and stereotypies in the same patient population. We therefore set out to conduct a systematic literature review to assess the relationship between tics and stereotypies when these conditions present in co-morbidity in otherwise typically developing subjects.

## Methods

The present systematic literature review was conducted in accordance with the Preferred Reporting Items for Systematic Reviews and Meta-Analyses (PRISMA) guidelines [16], used in conjunction with the Explanation and Elaboration document [17]. And the PRISMA-S extension to the PRISMA Statement for Reporting Literature Searches in Systematic Reviews [18]. We performed a search using MEDLINE via the PubMed interface for original studies on patients presenting with both tics and stereotypies. For our search, we combined the terms ‘tic disorder’ OR ‘Tourette’ with ‘stereotyp*’. Moreover, the reference lists of eligible articles were screened to identify any further relevant articles. For comprehensiveness, we manually searched the archives of the scientific journals where the eligible articles were published. We excluded from our review smaller studies with a sample size inferior to 40 patients, as well as studies on patients with an underlying diagnosis of a pervasive neurodevelopmental disorder, such as autism spectrum disorder. Finally, we limited our search to original studies published in the English language, but there were no chronological, geographic or demographic limitations to the inclusion of articles.

## Results

**Fig. 1:**
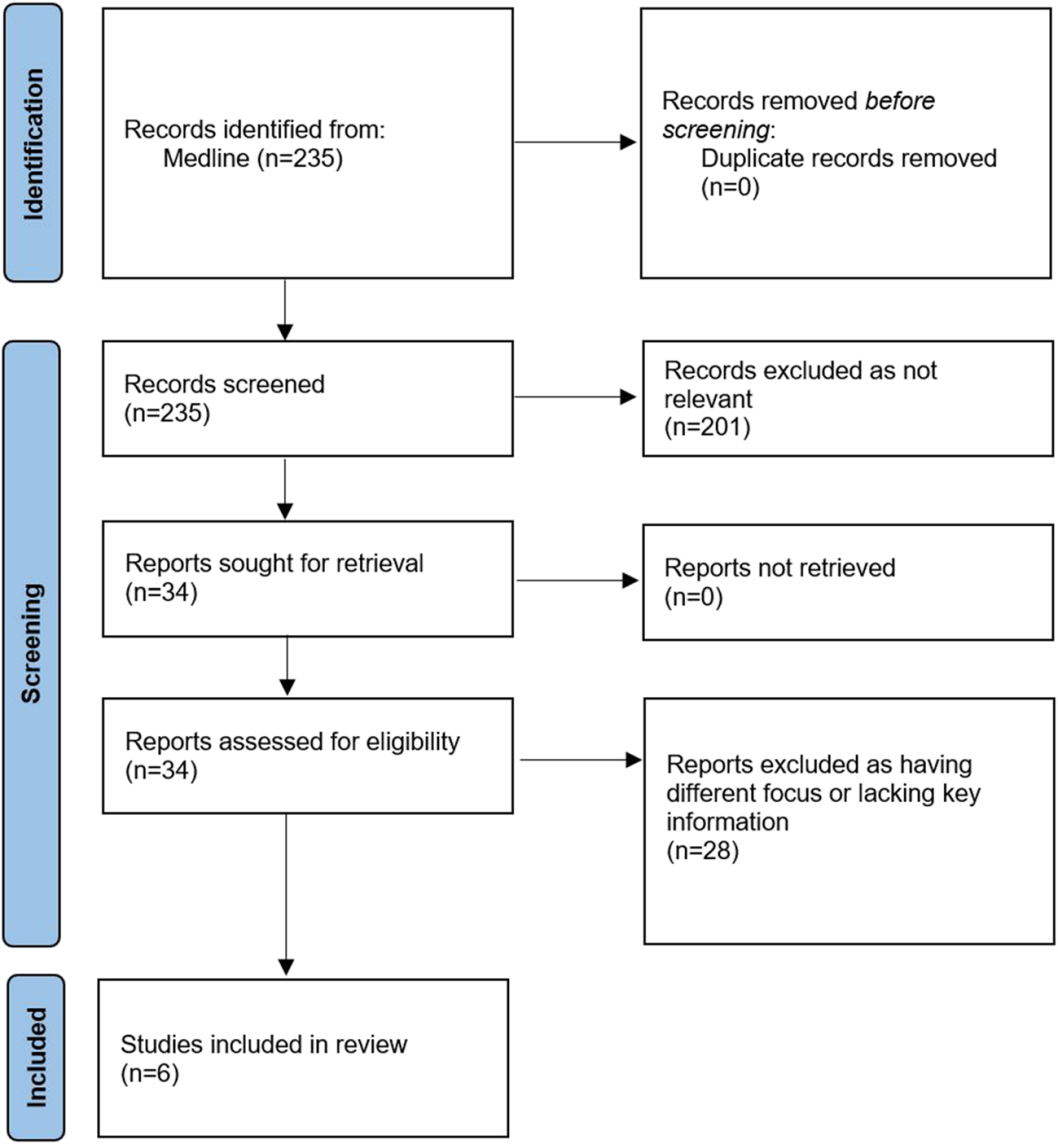
PRISMA flow diagram outlining the identification, screening, assessment for eligibility, and inclusion of studies in the present review

Our systematic literature search yielded a total of 235 articles, without any duplicates. Of these, 34 were considered relevant to the review and their full texts were inspected. A further 28 studies were excluded because they had a different focus or lacked key information. A total of six articles fulfilled our criteria and were included in the present review. The article selection process is summarised in the PRISMA flow diagram (Fig. 1).

The reviewed articles included four studies of patients who presented with tics in the context of stereotypic movement disorder (*n* = 231, mean ages ranging from 8 to 13 years) [15, 19–21], and two studies of patients who presented with stereotypies in the context of Tourette syndrome (*n* = 344, mean age 30 years) [22, 23]. All the studies were conducted on otherwise typically developing subjects seen in specialist clinics between 2004 and 2021 (Table 1).

**Table 1:** Summary of studies on co-morbid tics and stereotypies (*n* ≥ 40)

Study	Clinical population	n	Mean age	Male gender	Tics	Stereotypies	ADHD	OCS
Mahone et al. 2004 [19]	Stereotypic movement disorder	40	8 years	25 (63%)	7 (18%)	40 (100%)	10 (25%)	4 (10%)
Harris et al. 2008 [20]	Stereotypic movement disorder	100	8 years	62 (62%)	18 (18%)	100 (100%)	30 (30%)	12 (12%)
Freeman et al. 2010 [15]	Stereotypic movement disorder	42	11 years	31 (73%)	18 (43%)	42 (100%)	16 (38%)	5 (12%)
Oakley et al. 2015 [21]	Stereotypic movement disorder	49	13 years	31 (63%)	11 (22%)	49 (100%)	31 (63%)	17 (35%)
Ubhi et al. 2020 [22]	Tourette syndrome	145	30 years	102 (70%)	145 (100%)	17 (12%)	35 (24%)	70 (48%)
Baizabal-Carvallo et al. 2021 [23]	Tourette syndrome	199	N/A	N/A	199 (100%)	11 (6%)	N/A	N/A

Abbreviations. *ADHD* Attention-deficit and hyperactivity disorder; *OCS* Obsessive-compulsive symptoms; *N/A* Not available

Overall, 23% (54/231) of patients diagnosed with stereotypic movement disorder presented with co-morbid tics. There was a considerable variability across the reviewed studies (range 18–43%). Across all studies, the majority of patients were males (65%, range 62–73%). Co-morbid conditions were reported as follows: 38% (range 25–63%) attention-deficit and hyperactivity disorder and 16% (range 10–35%) obsessive–compulsive symptoms.

Stereotypies were present in about 8% (28/344) of patients diagnosed with Tourette syndrome. There was low variability between the two reviewed studies (range 6–12%). Both studies reported data on a few patients with tics and stereotypies who also had a diagnosis of autism spectrum disorder: for this reason, 2 patients were excluded from our analysis of the study by Baizabal-Carvallo and Jankovic [23] and 3 patients were excluded from our analysis of the study by Ubhi et al. [22]. Data about gender and co-morbidities were available only for the study by Ubhi et al. [22], where the majority of patients were males (70%) and the co-morbidity rates of attention-deficit and hyperactivity disorder and obsessive–compulsive symptoms were 24% and 48%, respectively.

## Discussion

Our literature search identified six original studies presenting data on the association between tics and stereotypies in otherwise typically developing patients of paediatric age. The combined findings of our systematic literature review show that tics and stereotypies can often develop in co-morbidity. Specifically, we found that up to one in four patients diagnosed with stereotypic movement disorder present with co-morbid tics, and up to one in twelve patients with Tourette syndrome presents with both tics and stereotypies. Of note, we found a striking gender imbalance, as 65–70% of patients presenting with both tics and stereotypies across the reviewed studies were males. This is consistent with previous data from the literature on both Tourette syndrome and stereotypic movement disorder. It has consistently been reported that tics are at least three times more common in males than in females [3]. Likewise, several reports have suggested a higher rate of motor stereotypies in males as compared with females, with an approximate 3:2 ratio [24].

The four studies focusing on young patients who present with tics in the context of stereotypic movement disorder found a wide range of tic prevalence Figs. (18–43%). Combined data from our literature review show that around 23% of patients with stereotypies also have tics. This is at the upper end of the range of prevalence figures for tics in childhood (4 to 24%) reported in the general population [25]. High rates of other co-morbidities were noted across the reviewed studies, with an average of 38% of patients also presenting with attention-deficit and hyperactivity disorder, and 16% with obsessive–compulsive symptoms. Such co-morbidity profile reflects the common observations that stereotypies are frequently associated with other neurodevelopmental conditions and – to a lesser extent – other repetitive behaviours [26].

It is widely acknowledged that stereotypies may be present in otherwise typically developing children as a benign movement disorder [27], although they can become persistent [28]. However, the frequency and impact of motor stereotypies is considerably higher in patients with autism spectrum disorder, with prevalence rates ranging from 22 to 98% [29]. Based upon clinical experience, it has been highlighted that stereotypies in patients with stereotypic movement disorder are not distinguishable from those in patients with autism spectrum disorder solely by their motor description [15, 30]. Specific alterations in social cognition and communication associated with autism spectrum disorder, along with measures of global functioning, are helpful features in the differential diagnosis of doubtful cases [31]. It has been suggested that tics can often be co-morbid with stereotypies in the context of pervasive developmental disorders. In five boys diagnosed with both Asperger’s disorder and Tourette syndrome, history was notable for a flapping stereotypy, as well as clumsiness and exceptional verbal intelligence [32]. In a case series of 12 patients with autism spectrum disorders referred to a specialist movement disorders clinic for the evaluation of tics, all patients exhibited stereotypic movements and seven of them had tics [33]. Analysis of video sequences of 21 adults with autism spectrum disorder and a repertoire of 10 different stereotypies (57% males, mean age 35 years) revealed the presence of co-morbid motor tics in half of the patients [34]. More recently, in an expert video-based evaluation of 27 patients with a diagnosis of severe autism spectrum disorder with stereotypies (89% males, mean age 14 years), there was evidence of possible tics in 67% of the clinical sample [35]. These findings suggest the possibility of a considerably higher rate of co-morbid tics in this patient population, however there is a need to integrate reviews of video material with a comprehensive clinical assessment, focusing on the diagnostic re-evaluation of heterogeneous motor manifestations. Finally, the association between tics and stereotypies has been reported in a number of other neuropsychiatric conditions characterised by behavioural and cognitive abnormalities, such as Rett syndrome [36], Primrose syndrome [37], a juvenile progressive dystonia suggestive of pantothenate kinase-associated neurodegeneration (PKAN) [38], and behavioural disturbance associated with anti-basal ganglia antibodies [39].

It has been estimated that about a third of patients with Tourette syndrome present with co-morbid movement disorders, which should be differentiated and distinguished from tics based on their clinical phenomenology, etiopathogenesis, and response to treatment interventions [23]. The combined results of our systematic literature review confirm that stereotypies are among the most common movement disorders diagnosed in patients with tic disorders. Specifically, we identified two large studies recently conducted in adult specialist movement disorders clinics that reported a combined prevalence of primary stereotypies of 8%, with a narrow range of variability. Of note, both studies reported data on a few other patients with tics and stereotypies who also had a diagnosis of autism spectrum disorder, which were therefore excluded from our analysis. Taking into consideration the relatively high prevalence of pervasive developmental disorders in patients with Tourette syndrome, the 8% figure for co-morbid stereotypies in this clinical population might be an underestimate. This figure is at the upper limit of the prevalence estimates for primary complex motor stereotypies in the general population, which are reported to range between 3 and 8% [21, 40]. Overall, the high rates of co-occurrence between tics and stereotypies are consistent with the available evidence on shared pathophysiological mechanisms including basal ganglia networks [1]. Specifically, altered striatal function mainly involving dopaminergic pathways has been reported in animal models of both tics [41] and stereotypies [42], suggesting that the ‘striatal disinhibition model’ could inform our understanding of both conditions [43, 44].

Leg stereotypy syndrome was recently proposed as a common type of stereotypy typically beginning in childhood and presenting with repetitive movements affecting almost exclusively the legs while the patient is seated (and both the legs and the trunk while standing) [45]. In a study on 13 patients with Tourette syndrome seen at a specialist movement disorders clinic, leg stereotypy syndrome was observed in 30% of patients, compared to 7% of controls [46]. When prompted to focus on the subjective experiences accompanying their repetitive movements, most adult patients are able to clearly differentiate between their tics and stereotypies, as the latter ones lack the characteristic premonitory urge to tic. The absence of correlation between stereotypy severity measures and tic severity ratings in the study by Ubhi et al. [22] provides further evidence in support of the clinical distinction between stereotypies and tics. Moreover, the co-morbidity profile of patients with Tourette syndrome and stereotypies was characterised by a higher prevalence of tic-related obsessive–compulsive symptoms as compared to patients with a primary diagnosis of stereotypic movement disorder.

Our systematic literature review has limitations. First, all participants were recruited from specialist centres, where patients with more severe clinical presentations and multiple co-morbidities can be over-represented (referral bias). Second, the differential diagnosis between complex motor tics and stereotypies can be notoriously challenging, however in the reviewed studies motor abnormalities were consistently assessed by movement disorder specialists. Third, there was a discrepancy in the age group between the studies that focused on co-morbid tics in patients with stereotypic movement disorder (paediatric age) and studies that focused on co-morbid stereotypies in patients with Tourette syndrome (adult age). In consideration of the relative paucity of research on co-occurring tics and stereotypies in otherwise typically developing subjects, studies focusing on paediatric and adult populations were combined in the present review: this might have affected the overall results according to the different clinical presentations and assessment protocols across the lifespan. Finally, patients with autism and severe learning disability were excluded from our systematic literature review, possibly resulting in underestimation of the prevalence of co-morbid stereotypies, since they are included in the current diagnostic criteria for autism spectrum disorders.

In conclusion, the available evidence indicates that tics and stereotypies can often develop in co-morbidity. The association of tics and stereotypies in the same patient has practical implications, in consideration of the different treatment approaches. Specifically, a better understanding of the clinical presentation of stereotypies in patients with tic disorders can help preventing the misdiagnosis of these symptoms as refractory tics, as well as the implementation of inappropriate treatment interventions. The possibility that treatment-refractory repetitive movements in adults with Tourette syndrome could represent persistent stereotypies should be taken into account. From an experimental perspective, the ability to differentiate tics from stereotypies within the same subject could help addressing some of the challenges associated with the use of animal models for the study of the neurodevelopmental abnormalities underlying repetitive motor behaviours [47].

Future research should focus on the assessment and management of patients presenting with both conditions, particularly in special populations (e.g. patients with pervasive developmental disorders and/or intellectual disability) [28]. In addition to epidemiological studies in the general community, there is a need for clinical studies focusing on the prevalence of stereotypies in patients with tics attending paediatric neurology and child and adolescent psychiatry clinics. Specifically, cross-sectional studies on larger samples of children and adults would be both feasible and informative. Longitudinal studies could provide useful data on the natural course of co-occurring tics and stereotypies across the lifespan. It would be useful to further characterise stereotypies in young patients with tic disorders in order to identify discrete phenomenological subgroups. These include – but are not limited to – intense imagery movements, a common and distinct paediatric subgroup of motor stereotypies where children engage in acts of imagery while performing stereotyped movements [48, 49]. Ultimately, a better understanding of the role played by altered physiological states, such as increased arousal [50], could pave the way to the development of more targeted treatment interventions for both stereotypies and tics, ranging from behavioural approaches to pharmacotherapy.

## References

[1] Martino D, Hedderly T (2019). Tics and stereotypies: a comparative clinical review. Parkinsonism Relat Disord.

[2] American Psychiatric Association (2022) Diagnostic and statistical manual of mental disorders, 5th edn., text revision. American Psychiatric Association Publishing, Washington

[3] Martino D, Madhusudan N, Zis P, Cavanna AE (2013). An introduction to the clinical phenomenology of Tourette syndrome. Int Rev Neurobiol.

[4] Singer HS (2009). Motor stereotypies. Semin Pediatr Neurol.

[5] Singer HS (2013). Motor control, habits, complex motor stereotypies, and Tourette syndrome. Ann N Y Acad Sci.

[6] Edwards MJ, Lang AE, Bhatia KP (2012). Stereotypies: a critical appraisal and suggestion of a clinically useful definition. Mov Disord.

[7] Martino D, Cavanna AE, Robertson MM, Orth M (2012). Prevalence and phenomenology of eye tics in Gilles de la Tourette syndrome. J Neurol.

[8] Cox JH, Seri S, Cavanna AE (2018). Sensory aspects of Tourette syndrome. Neurosci Biobehav Rev.

[9] Cavanna AE (2022). Current and emerging pharmacotherapeutic strategies for Tourette syndrome. Expert Opin Pharmacother.

[10] Bhatoa RS, Malik O, Robinson S, Hedderly T (2021) Clinical management of complex motor stereotypies. Arch Dis Child:322624. 10.1136/archdischild-2021-322624 10.1136/archdischild-2021-322624 34725047

[11] Cavanna AE (2018). Gilles de la Tourette syndrome as a paradigmatic neuropsychiatric disorder. CNS Spectr.

[12] Brierley NJ, McDonnell CG, Parks KMA, Schulz SE, Dalal TC, Kelley E, Anagnostou E, Nicolson R, Georgiades S, Crosbie J, Schachar R, Liu X, Stevenson RA (2021). Factor structure of repetitive behaviors across autism spectrum disorder and attention-deficit/hyperactivity disorder. J Autism Dev Disord.

[13] Neal M, Cavanna AE (2013). "Not just right experiences" in patients with Tourette syndrome: complex motor tics or compulsions?. Psychiatry Res.

[14] Eddy CM, Cavanna AE (2014). Tourette syndrome and obsessive compulsive disorder: compulsivity along the continuum. J Obsessive Compuls Relat Disord.

[15] Freeman RD, Soltanifar A, Baer S (2010). Stereotypic movement disorder: easily missed. Dev Med Child Neurol.

[16] Page MJ, McKenzie JE, Bossuyt PM, Boutron I, Hoffmann TC, Mulrow CD, Shamseer L, Tetzlaff JM, Akl EA, Brennan SE, Chou R, Glanville J, Grimshaw JM, Hróbjartsson A, Lalu MM, Li T, Loder EW, Mayo-Wilson E, McDonald S, McGuinness LA, Stewart LA, Thomas J, Tricco AC, Welch VA, Whiting P, Moher D (2021). The PRISMA 2020 statement: an updated guideline for reporting systematic reviews. BMJ.

[17] Page MJ, Moher D, Bossuyt PM, Boutron I, Hoffmann TC, Mulrow CD, Shamseer L, Tetzlaff JM, Akl EA, Brennan SE, Chou R, Glanville J, Grimshaw JM, Hróbjartsson A, Lalu MM, Li T, Loder EW, Mayo-Wilson E, McDonald S, McGuinness LA, Stewart LA, Thomas J, Tricco AC, Welch VA, Whiting P, McKenzie JE (2021). PRISMA 2020 explanation and elaboration: updated guidance and exemplars for reporting systematic reviews. BMJ.

[18] Rethlefsen ML, Kirtley S, Waffenschmidt S, Ayala AP, Moher D, Page MJ, Koffel JB, PRISMA-S Group (2021). PRISMA-S: an extension to the PRISMA Statement for Reporting Literature Searches in Systematic Reviews. Syst Rev.

[19] Mahone EM, Bridges D, Prahme C, Singer HS (2004). Repetitive arm and hand movements (complex motor stereotypies) in children. J Pediatr.

[20] Harris KM, Mahone EM, Singer HS (2008). Nonautistic motor stereotypies: clinical features and longitudinal follow-up. Pediatr Neurol.

[21] Oakley C, Mahone EM, Morris-Berry C, Kline T, Singer HS (2015). Primary complex motor stereotypies in older children and adolescents: clinical features and longitudinal follow-up. Pediatr Neurol.

[22] Ubhi M, Achinivu K, Seri S, Cavanna AE (2020). Motor stereotypies in adult patients with Tourette syndrome. Future Neurol.

[23] Baizabal-Carvallo JF, Jankovic J (2021). Beyond tics: movement disorders in patients with Tourette syndrome. J Neural Transm.

[24] Muthugovindan D, Singer H (2009). Motor stereotypy disorders. Curr Opin Neurol.

[25] Robertson MM (2015). A personal 35 year perspective on Gilles de la Tourette syndrome: prevalence, phenomenology, comorbidities, and coexistent psychopathologies. Lancet Psychiatry.

[26] Barry S, Baird G, Lascelles K, Bunton P, Hedderly T (2011). Neurodevelopmental movement disorders - an update on childhood motor stereotypies. Dev Med Child Neurol.

[27] Bonnet C, Roubertie A, Doummar D, Bahi-Buisson N, Cochen de Cock V, Roze E (2010). Developmental and benign movement disorders in childhood. Mov Disord.

[28] Tan A, Salgado M, Fahn S (1997). The characterization and outcome of stereotypical movements in nonautistic children. Mov Disord.

[29] Melo C, Ruano L, Jorge J, Pinto Ribeiro T, Oliveira G, Azevedo L, Temudo T (2020). Prevalence and determinants of motor stereotypies in autism spectrum disorder: a systematic review and meta-analysis. Autism.

[30] Ghanizadeh A (2010). Clinical approach to motor stereotypies in autistic children. Iran J Pediatr.

[31] Mandy WP, Skuse DH (2008). Research review: what is the association between the social-communication element of autism and repetitive interests, behaviours and activities?. J Child Psychol Psychiatry.

[32] Nass R, Gutman R (1997). Boys with Asperger’s disorder, exceptional verbal intelligence, tics, and clumsiness. Dev Med Child Neurol.

[33] Ringman JM, Jankovic J (2000). Occurrence of tics in Asperger’s syndrome and autistic disorder. J Child Neurol.

[34] Kahl U, Schunke O, Schöttle D, David N, Brandt V, Bäumer T, Roessner V, Münchau A, Ganos C (2015). Tic phenomenology and tic awareness in adults with autism. Mov Disord Clin Pract.

[35] Termine C, Grossi E, Anelli V, Derhemi L, Cavanna AE (2021). Possible tics diagnosed as stereotypies in patients with severe autism spectrum disorder: a video-based evaluation. Neurol Sci.

[36] Temudo T, Freitas P, Sequeiros J, Maciel P, Oliveira G (2008). Atypical stereotypies and vocal tics in Rett syndrome: an illustrative case. Mov Disord.

[37] Dalal P, Leslie ND, Lindor NM, Gilbert DL, Espay AJ (2010). Motor tics, stereotypies, and self-flagellation in Primrose syndrome. Neurology.

[38] Nardocci N, Rumi V, Combi ML, Angelini L, Mirabile D, Bruzzone MG (1994). Complex tics, stereotypies, and compulsive behavior as clinical presentation of a juvenile progressive dystonia suggestive of Hallervorden-Spatz disease. Mov Disord.

[39] Edwards MJ, Dale RC, Church AJ, Trikouli E, Quinn NP, Lees AJ, Giovannoni G, Bhatia KP (2004). Adult-onset tic disorder, motor stereotypies, and behavioural disturbance associated with antibasal ganglia antibodies. Mov Disord.

[40] MacDonald R, Green G, Mansfield R, Geckeler A, Gardenier N, Anderson J, Holcomb W, Sanchez J (2007). Stereotypy in young children with autism and typically developing children. Res Dev Disabil.

[41] Pogorelov V, Xu M, Smith HR, Buchanan GF, Pittenger C (2015). Corticostriatal interactions in the generation of tic-like behaviors after local striatal disinhibition. Exp Neurol.

[42] Canales JJ, Graybiel AM (2000). A measure of striatal function predicts motor stereotypy. Nat Neurosci.

[43] Worbe Y, Lehericy S, Hartmann A (2015). Neuroimaging of tic genesis: present status and future perspectives. Mov Disord.

[44] Mahone EM, Crocetti D, Tochen L, Kline T, Mostofsky SH, Singer HS (2016). Anomalous putamen volume in children with complex motor stereotypies. Pediatr Neurol.

[45] Jankovic J (2016). Leg stereotypy disorder. J Neurol Neurosurg Psychiatry.

[46] Lotia M, York MK, Strutt AM, Jankovic J (2018). Leg stereotypy syndrome: phenomenology and prevalence. J Neurol Neurosurg Psychiatry.

[47] Bortolato M, Pittenger C (2017). Modeling tics in rodents: conceptual challenges and paths forward. J Neurosci Methods.

[48] Robinson S, Woods M, Cardona F, Baglioni V, Hedderly T (2014). Intense imagery movements: a common and distinct paediatric subgroup of motor stereotypies. Dev Med Child Neurol.

[49] Robinson S, Woods M, Cardona F, Hedderly T (2016). Intense Imagery Movements (IIM): more to motor stereotypies than meets the eye. Eur J Paediatr Neurol.

[50] Nagai Y, Cavanna A, Critchley HD (2009). Influence of sympathetic autonomic arousal on tics: implications for a therapeutic behavioral intervention for Tourette syndrome. J Psychosom Res.

